# Differences in growth patterns and catch-up growth of small for gestational age preterm infants fed on fortified mother’s own milk *v*. preterm formula

**DOI:** 10.1017/S0007114522000599

**Published:** 2023-06-28

**Authors:** Lilach Hofi, Orna Flidel-Rimon, Calanit Hershkovich–Shporen, Hilla Zaharoni, Ruth Birk

**Affiliations:** 1 Department of Nutritional Sciences, School of Health Sciences, Ariel University, Ariel, Israel; 2 Department of Neonatology, Kaplan Medical Center, Rehovot, Hebrew University, Jerusalem, Israel; 3 Department of Clinical Nutrition, Kaplan Medical Center, Rehovot, Hebrew University, Jerusalem, Israel

**Keywords:** Mother’s own milk, Preterm formula, Small for gestational age, Preterm infants, Growth

## Abstract

Small for gestational age (SGA) is typically defined as birth weight < 10th percentile for age. Limited data are available regarding the growth of SGA preterm infants in relation to feeding type. We aimed to study SGA preterm infants fed fortified mother’s own milk (MOM) or preterm formula (PF) on growth patterns and catch-up growth at discharge and 2-year corrected age (CA). Our retrospective cohort study included data from medical records and follow-up questionnaires about SGA preterm infants born at < 37 weeks fed on MOM (*n* 40) and PF (*n* 40). Weight, length/height and head circumference (HC) were collected at birth, discharge and at 2-year CA, and Δ z-scores were calculated. The MOM group had significantly larger negative change in weight and length z-scores between birth and discharge, and smaller positive change in HC z-score (–0·47 (sd 0·41) *v.* −0·25 (sd 0·36), *P* = 0·01; −0·63 (sd 0·75) *v.* −0·27 (sd 0·75), *P* = 0·03; 0·13 (sd 0·67) *v*. 0·41 (sd 0·55), *P* = 0·04, respectively). Almost half of the MOM-fed infants experienced poor length growth by discharge compared with 22 % of PF-fed infants (*P* = 0·03). By 2-year CA, both groups had similar positive change in weight and HC z-scores, but MOM-fed infants had a slower increase in height z-score (0·64 (sd 1·30) *v*. 1·33 (sd 1·33), *P* = 0·02), and only 40 % had achieved catch-up height compared with 68 % of the PF group (*P* = 0·02). Our study indicates that fortified MOM-fed SGA preterm infants may need extra nutritional support in the first 2 years of life to achieve height growth potential.

Globally, 15 million babies are born preterm annually, a preterm birth rate of ∼11 %.

Of these infants, ∼20 % are born small for gestational age (SGA)^(1)^. SGA is typically defined as birth weight < 10th percentile for age. Others define SGA as birth weight below the 3rd or 5th percentile, or less than –2 sd of expected birth weight for gestational age (GA)^(2–4)^. Fetal growth restriction occurs due to several factors, including placental insufficiency, genetics, inadequate nutrition, deprived social-environmental states and maternal health issues^(4)^. SGA preterm infants have a two to fourfold elevated risk of mortality compared with non-SGA preterm infants. Moreover, they are prone to multisystem complications, such as anaemia, respiratory failure, necrotising enterocolitis, retinopathy of prematurity, bronchopulmonary dysplasia and patent ductus arteriosus^(4,5)^.

Nutrition plays an essential role in the first 1000 d of a preterm infant’s life, with critical health consequences on growth, morbidity and cognitive development^(4)^. Mother’s own milk (MOM) is the first choice to feed preterm infants^(4–6)^, the advantages include improved immune defence and gastrointestinal function, reduction in the incidence of necrotising enterocolitis and sepsis, and improved long-term neurodevelopment outcomes compared to feeding with preterm formula (PF)^(7,8)^. However, preterm infants’ nutritional needs are beyond what is provided in human milk^(9)^. Therefore, fortifying human milk with a human milk fortifier is recommended for preterm infants with a birth weight < 1800 g^(10)^. When MOM and donor milk are not available, the recommendation is to feed preterm infants with PF that supply has a higher nutrient density than standard infant formula. PF appear to match the nutritional needs of preterm infants, and they support good growth^(11)^; however, limited data are available regarding growth patterns of SGA preterm infants.

Our aim was to study growth patterns of SGA preterm infants fed fortified MOM or PF at two time points: discharge and 2-year corrected age (CA) follow-up.

## Experimental methods

### Study design

This is a retrospective cohort study conducted during the year 2020, using data collected from medical records of SGA preterm infants born at Kaplan Medical Center’s (KMC) Neonatal Intensive Care Unit (NICU) between the years 2012–2018.

Anthropometric data at 2-year CA were extracted from KMC’s neonatal follow-up clinic medical records and from family healthcare centres, subject to parental consent. In addition, a follow-up telephone questionnaire was conducted with parents in 2020(average age of the children in 2020 was 4·8 years). The questionnaire included standard questions that are also asked at the Israeli follow-up health clinics, such as information regarding duration of breast-feeding, type of formula and timing of introduction of complementary food.

This study was conducted according to the Declaration of Helsinki guidelines, and all procedures involving human were approved by the KMC’s Ethics Committee (0130-19-KMC). Verbal informed consent was obtained from all parents.

### Study population

A total of 191 SGA preterm infants were born at KMC between January 2012 and December 2018 with GA < 37 + 0 weeks. Inclusion criteria included SGA preterm infants fed MOM or PF. SGA was defined as per Fenton’s growth chart^(12)^. Exclusion criteria included mixed feeding (MOM ≤ 80 % or PF ≤ 80 % of daily volume) (*n* 89), lack of matching (*n* 10), mortality (*n* 5), congenital malformations (*n* 4) and genetic syndrome (*n* 3). After applying the exclusion and inclusion criteria, we included eighty SGA preterm infants and excluded 111 SGA preterm infants. Using MedCalc software, the sample size was calculated to be at least seventy SGA preterm infants, thirty-five in each group with 80 % power and a significance level of *P*-value < 0·05 to detect 50 % difference between groups.

**Fig. 1.**
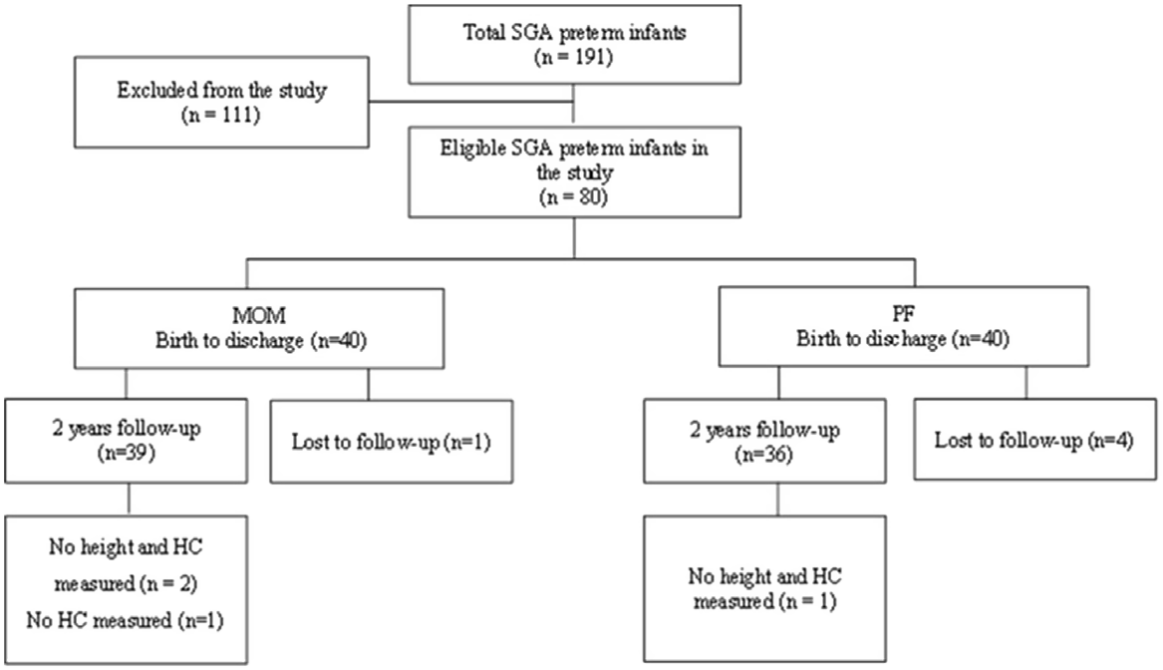
Study flow chart. SGA, small for gestational age; MOM, mother’s own milk; PF, preterm formula; HC, head circumference.

Feeding type was defined based on standardised reporting of neonatal nutrition and growth (StRoNNG)^(13)^. We divided the data into two groups: the MOM group included forty preterm infants primarily fed with MOM (volume of feeds > 80 %) and the PF group included forty preterm infants primarily fed with PF (volume of feeds > 80 %). Each preterm infant from the MOM group was matched to a preterm infant from the PF group. The criteria were birth week (sd1 week), birth weight (sd100 g) and birth year. Follow-up data, including anthropometric measures at 2-year CA and eligible questionnaires, were available for seventy-five infants (see flow chart in Fig. 1).

### Outcome measures and definitions

Anthropometric measures, including infant weight, length/height and head circumference (HC), were collected at birth, discharge and 2-year CA according to StRoNNG^(13)^.

Anthropometric growth data were converted to z-scores according to standardised age at assessment and infant sex. Changes in z-scores (Δ z-score) were calculated from birth to discharge using Fenton’s growth chart (12), and from birth to the 2-year follow-up, using the WHO growth chart (https://www.who.int/tools/child-growth-standards/standards)^(14)^.

According to standardised procedures, weight was measured to the nearest 5 g using a calibrated electronic scale (Shekel Scale LTD, serial 502449), and length was measured with a recumbent length board measured to the nearest 1 mm (Yogel Electronics Ltd, MR-100) and HC with a non-stretchable tape measure. All measurements were done with standardised equipment and techniques by professionals that were trained in measuring preterm infants and toddlers. Expected growth was defined as the change in z-score on the Fenton growth chart between −0·8 and 0·8 from birth to discharge^(15)^. We also classified poor weight growth and malnutrition as a decline of > 0·8 sd in weight-for-age z-scores, poor linear growth and malnutrition as a decline of > 0·8 sd in length-for-age z-score and poor HC growth as a decline of > 1 sd in HC-for-age z-score^(16–19)^. Rapid weight growth was defined as Δ z-score > 1 sd at discharge^(20)^. Catch-up growth in weight, height or HC at 2-year CA was defined as Δ z-score > 0·67 sd ^(21,22)^. Weight gain velocity g/kg/day was calculated according to the equation: [(W2 − W1)/([(W2 + W1)/2]/1000)/number of days]^(23)^. Clinical and demographic data such as maternal age, delivery mode, maternal morbidities, ethnicity, intra-uterine growth restriction, day of full enteral feeding, postnatal weight loss, day of birth weight regain, days of hospitalisation and neonatal morbidities were also extracted from medical records.

### Feeding protocol

The KMC’s NICU follows a feeding protocol based on the European Society for Paediatric Gastroenterology, Hepatology and Nutrition’s (ESPHAGAN) recommendations of parenteral and enteral + nutrition^(24–27)^. Trophic feeding (< 24 ml/kg per d) was initiated within the first 24 h of life and no later than 48 h, with advancements of 20–35 ml/kg per d until full enteral feed was reached. To meet the nutritional needs, MOM was fortified by bovine milk-based human milk fortifier powder (one packet /14·644 kJ (3·5 kcal), 0·25 g protein; Similac Abbott Nutrition). Fortification was introduced when MOM feeding exceeded 50–80 ml/kg/d, starting with two packets of human milk fortifier/100 ml (22 kcal/oz) and advanced to four packets of human milk fortifier/100 ml (24 kcal/oz) when reaching 100 ml/kg per d. PF preterm infants were fed (80 kcal, 2·6–2·88 g protein per 100 ml). Decisions regarding feeding volume and fortification were made by the physician and dietitian, based on the feeding guideline. The goals for enteral nutrition were 140–160 ml/kg per d, 120–130 kcal/kg per d and 3·5–4 g protein/kg per d. All the SGA preterm infants in the study received 150–160 ml/kg per d and achieved the nutritional goals as they reached full enteral feeding.

### Data analysis

Data are presented as mean values and standard deviations or numbers and percentages. Continuous variables between the study groups were tested for normality by a Shapiro–Wilk test. Since normal distributions were found, and T-tests were performed for continuous variables. Fisher’s exact tests were used to test the relationship between two categorical variables. A Pearson correlation two-tailed was preformed between day of regain and Δ height z-score at 2-year CA. *P* values < 0·05 were considered statistically significant, 95 % confidence interval, with 80% power. Data were analysed using the Statistical Package for the Social Sciences (SPSS, software version 25).

## Results

Eighty SGA preterm infants were included in this study (forty MOM fed and forty PF fed). The baseline characteristics of the SGA preterm infants and their respective mothers are presented in Table 1. No differences were found in the baseline characteristics of the two groups, such as anthropometric measurements, morbidity, supplemented oxygen, postnatal weight loss (%), day of weight nadir, day of birth weight regain, day of full enteral feeding and days of hospitalisation. In addition, no differences were found in maternal characteristics including ethnicity and maternal morbidity (data not shown).

**Table 1.** Characteristics and comparison of nutrition groups* (Mean values and standard deviations; numbers and percentages)

	MOM (*n* 40)			PF (*n* 40)			
Neonatal characteristics		Mean	sd		Mean	sd	*P*
Male							0·17
*n*	25			18			
%	62			45			
Gestational age (week)		33·8	2·4		34·7	1·9	0·06
Birth weight (g)		1409	319		1490	310	0·25
Birth weight z-score (sd)		–2·04	0·53		–2·28	0·56	0·06
Birth length (cm)		39·6	3·8		39·7	3·4	0·85
Birth length z-score (sd)		–1·81	0·99		–2·15	0·89	0·11
Birth head circumference (cm)		28·7	2·4		29·1	2·1	0·48
Birth head circumference z-score (sd)		–1·34	0·96		–1·60	0·78	0·20
Multiple births							0·009‡
*n*	8			20			
%	20			50			
Postmenstrual age at discharge (week)		38·1	1·3		38·4	1·6	0·44
Intra-uterine growth restriction							0·48
*n*	28			24			
%	70			60			
SGA asymmetric							0·81
*n*	17			15			
%	42			37			
Postnatal weight loss (%)		3·9	2·7		3·1	2·2	0·15
Day of weight nadir		1·4	1·4		1	0·9	0·11
Weight at nadir (g)		1355	314		1446	307	0·19
Day of weight regain		4·9	2·6		4·3	2·1	0·26
Day of full enteral feeding (≥140 ml/kg)		4·8	1·6		5·6	2·2	0·10
Episodes of regurgitation during hospitalisation		2	2·5		5·9	8·5	0·007‡
Necrotising enterocolitis							
*n*	0						1·00
Intra-ventricular haemorrhage							0·49
*n*	0			2			
%				5			
Bronchopulmonary dysplasia							0·35
*n*	4			1			
%	10			2			
Patent ductus arteriosus							0·67
*n*	4			2			
%	10			5			
Late onset sepsis							1·00
*n*	1			1			
%	2			2			
Supplemented oxygen							1·00
*n*	14			14			
%	35			35			
Days of hospitalisation		30·8	22·1		26	18·6	0·28
Maternal characteristics							
Maternal age (years)		32·7	6		32·4	5·3	0·80
Spontaneous pregnancy							0·13
*n*	32			25			
%	80			62			
Vaginal delivery							0·42
*n*	7			11			
%	17			27			
Preeclampsia							0·19
*n*	7			13			
%	17			32			
Maternal diabetes							0·35
*n*	4			1			
%	10			2			

MOM, mother’s own milk; PF, preterm formula.

Mean ± sd, independent samples *t* test *P* values.

Number (%), Fisher’s exact test *P* values.

* Statistically significant difference.

In the SGA preterm infants MOM group, 62 % were boys *v*. 45 % in the PF group (*P* = 0·17). GA in the MOM group was 33·8 (sd 2·4) compared with 34·7 (sd 1·9) in the PF group (*P* = 0·06), and birth weight was 1409 (sd 319) in the MOM group compared with 1490 (sd 310) in the PF group (*P* = 0·25). Significant difference was found between the feeding groups in multiple births (MOM: 20 % *v.* PF: 50 %, *P* = 0·009). We anticipated that the PF group would have more multiple births since mothers find it difficult to express milk for twins or triplets^(20)^. Furthermore, the MOM group had significantly fewer episodes of regurgitation during hospitalisation compared with the PF group (2 (sd 2·5) *v.* 5·9 (sd 8·5), respectively, *P* = 0·007).

### Growth outcomes at discharge

The growth outcomes at discharge are shown in Table 2. Feeding with fortified MOM was significantly associated with a larger negative Δ weight z-score between birth and discharge (MOM: −0·47 (sd 0·41) *v.* PF: −0·25 (sd 0·36), *P* = 0·01) and Δ length z-score (MOM: −0·63 (sd 0·75) *v.* PF: −0·27 (sd 0·75), *P* = 0·03). The MOM group had a significant smaller positive ΔHC z-score compared with the PF group (0·13 (sd 0·67) *v*. 0·41 (sd 0·55), respectively, *P* = 0·04). Absolute weight, length, HC and their z-scores were similar in both groups at discharge. The MOM group had weight gain (g/kg per d) equal to the PF group at discharge (*P* = 0·56).

**Table 2.** Growth outcomes at discharge (Mean values and standard deviations)

	MOM (*n* 40)		PF (*n* 40)		
Anthropometric measurement	Mean	sd	Mean	sd	*P*
Weight (g)	2108	177	2133	190	0·55
Weight z-score	–2·51	0·49	–2·53	0·51	0·85
Δ weight z-score	–0·47	0·41	–0·25	0·36	0·01*
Length (cm)	43·2	2·3	43·6	2·1	0·37
Length z-score	–2·44	0·78	–2·43	0·91	0·95
Δ length z-score	–0·63	0·75	–0·27	0·75	0·03*
HC (cm)	32·2	1·1	32·3	1	0·65
HC z-score	–1·21	0·69	–1·18	0·61	0·83
Δ HC z-score	0·13	0·67	0·41	0·55	0·04*
Weight gain (g/kg per d)	16·6	3·8	17	3·87	0·56

MOM, mother’s own milk; PF, preterm formula; HC, head circumference.

Mean ± sd, independent samples *t* test *P* values.

Δ z-score of weight, HC and length were calculated from birth to discharge.

* Statistically significant difference.

### Poor growth and neonatal malnutrition at discharge

Forty seven percentage of SGA preterm infants in the MOM group experienced poor length growth with a significant decline of > 0·8 sd in length-for-age z-score, compared with 22 % in the PF group (*P* = 0·03). No significant difference was found between groups for poor weight and HC growth (Table 3).

**Table 3.** Poor growth and neonatal malnutrition at discharge (Numbers and percentages)

Parameters	MOM (*n* 40)		PF (*n* 40)		*P*
	*n*	%	*n*	%	
Poor weight growth	8	20	4	10	0·34
Decline of < 0·8 sd in weight-for-age z-score					
Poor length growth	19	47	9	22	0·03*
Decline of < 0·8 sd in length-for-age z-score					
Poor HC growth	2	5	0		0·49
Decline of < 1 sd in HC-for-age z-score					

MOM, mother’s own milk; PF, preterm formula; HC, head circumference.

Number (%), Fisher’s exact test *P* values.

* Statistically significant difference.

### Growth outcomes at 2-year corrected age

At 2-year follow-up CA, all anthropometric parameters, including weight, height and HC, were similar in both the MOM and PF groups, except for Δ height z-score. The PF group achieved a significantly greater increase in Δ height z-score than the MOM group (1·33 (sd 1·3) *v.* 0·64 (sd 1·3), respectively, *P* = 0·02). However, regarding Δ weight z-score and Δ HC z-score, the MOM group accomplished similar increase to the PF group (Δ weight z-score; *P* = 0·09, Δ HC z-score; *P* = 0·09). Weight-for-height z-score was similar in both groups (*P* = 0·79) (Table 4).

**Table 4.** Growth outcomes at 2-year follow-up corrected age (Mean values and standard deviations)

	MOM (*n* 39)		PF (*n* 36)		
Anthropometric measurement	Mean	sd	Mean	sd	*P*
Weight (kg)	10·7	1·3	10·7	1·1	0·96
Weight z-score	–0·9	1	–0·78	0·96	0·6
Δ weight z-score	1·1	1·1	1·52	1	0·09
Height (cm)	83·7	3	84·1	3·3	0·67
Height z-score	–1·1	0·95	–0·9	1·08	0·45
Δ height z-score	0·64	1·3	1·33	1·33	0·02*
HC (cm)	47·1	1·9	46·9	1·4	0·63
HC z-score	–0·51	1·22	–0·53	0·88	0·93
Δ HC z-score	0·8	1·02	1·17	0·76	0·09
weight for height z-score	–0·44	1·05	–0·51	0·98	0·79
Introducing complementary foods (m)	6·1	1·4	7·8	3·2	0·004*

MOM, mother’s own milk; PF, preterm formula; HC, head circumference

Mean ± sd, independent samples *t* test *P* values.

For HC measurement, *n* 36 for MOM, and *n* 35 for PF.

For weight for length z-score, *n* 37 for MOM, and *n* 35 for PF.

Δ z-score of weight, HC and height were calculated from birth to 2-year corrected age.

* Statistically significant difference.

**Fig. 2.**
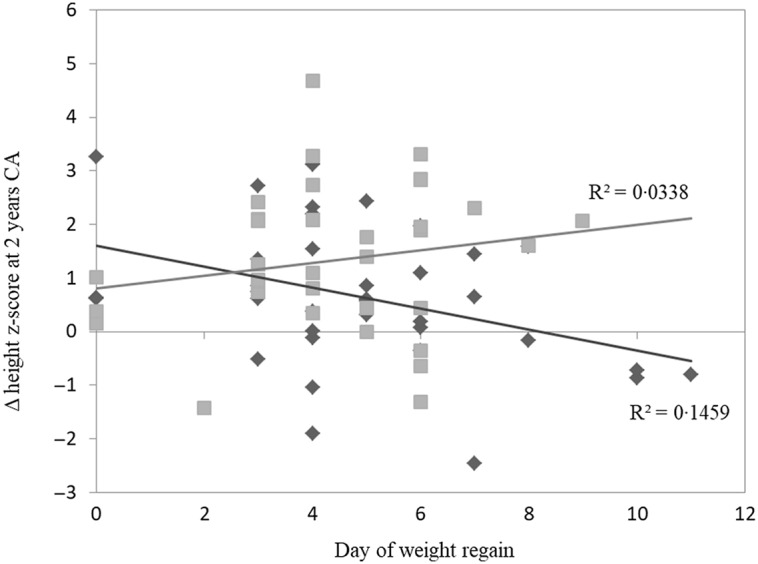
Correlation between day of weight regain and Δ height z-score from discharge to 2-year CA. MOM group had a significant inverse correlation with Δ height z-score at 2 years (*P* = 0·019). No correlation was found in PF group (*P* = 0·290). Pearson correlation two-tailed test. CA, corrected age; MOM, mother’s own milk; PF, preterm formula. 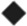, MOM; 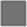, PF; 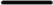, MOM; 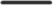, PF.

We also examined the correlation between the day of birth weight regain and the Δ height z-score at 2-year CA according to feeding type. The MOM group had a significant inverse correlation with Δ height z-score (*P* = 0·01); the longer it took SGA preterm infants to regain weight back to birth weight, the more it impacted their height growth over time. No correlation was found in the PF group (*P* = 0·29) (Fig. 2). After discharge, the preterm infants in the MOM group continued feeding with unfortified MOM for at least 3-month CA; 33 % of the MOM group continued breast-feeding for 3–6 months, 39 % for 6–12 months and 28 % for over a year. Furthermore, 69 % of the PF group were fed with post-discharge formula for at least 3-month CA and 31 % with standard formula after immediately discharge.

No difference was found between formula type and the infants’ growth outcome. The MOM group was introduced to complementary foods significantly earlier than the PF group (6·1 (sd 1·4) *v*. 7·8 (sd 3·2), *P* = 0·04). The mean age of the children at the time the parents answered the questionnaire was 5·1 (sd 1·6) years in the MOM group and 4·5 (sd 1·6) years in the PF group (*P* = 0·07).

### Catch-up growth at 2-year corrected age

Though not significant, 69 % of the MOM group infants had achieved weight catch-up growth by 2-year CA, compared with 86 % of the PF infants group (*P* = 0·10). A significant difference was found between feeding type and infants’ height outcome; 40 % of the MOM group achieved height catch-up growth compared with 68 % in the PF group (*P* = 0·02). The PF group also had a higher proportion of infants achieving HC catch-up growth (MOM: 43 % *v.* PF: 71 %, *P* = 0·05) (Table 5).

**Table 5.** Catch-up growths at 2-year follow-up corrected age (Numbers and percentages)

Parameters	MOM (*n* 39)		PF (*n* 36)		*P*
	*n*	%	*n*	%	
Weight catch-up growth	27	69	31	86	0·1
Δ weight z-score > 0·67 sd					
Height catch-up growth	15	40	24	68	0·02*
Δ height z-score > 0·67 sd					
HC catch-up growth	17	43	25	71	0·05*
Δ HC z-score > 0·67 sd					

MOM, mother’s own milk; PF, preterm formula; HC, head circumference.

Number (%), Fisher’s exact test *P* values.

For HC measurement, *n* 36 for MOM, and *n* 35 for PF.

For height measurement, *n* 37 for MOM, and *n* 35 for PF.

Δ z-score of weight, HC and height were calculated from birth to 2-year corrected age.

*P<0.05.

## Discussion

Our retrospective cohort study findings demonstrate that feeding SGA preterm infants with fortified MOM from birth is associated with significant twofold loss in weight and length z-scores at discharge compared with SGA preterm infants fed with PF. Furthermore, PF feeding is associated with a fourfold increase in HC z-scores compared to feeding with MOM at discharge.

SGA preterm feeding and growth consequences are challenging issue with complicated outcomes and scarcely studied. However, previously published findings regarding non-SGA preterm infants, type of feeding and growth parameters at discharge are partially consistent with our results. A small prospective observational study (*n* 32) on fortified MOM-fed non-SGA very low birth weight (VLBW) preterm infants found a significant decrease in weight z-score from birth to discharge compared with PF-fed non-SGA VLBW preterm infants. No differences were detected between the groups for Δ length or Δ HC z-scores^(28)^. Similarly, fortified-MOM or donor milk-fed non-SGA VLBW preterm infants (*n* 462, GA ≤ 32 week) were found to have significant reduction in weight z-score from birth to discharge compared with PF-fed preterm infants. Length and HC were not examined^(29)^. Contrary to these studies, a retrospective cohort study (*n* 466, GA < 37 weeks, birth weight < 2200 g) showed that low birth weight preterm infants fed with fortified breast milk (MOM and donor milk) had a higher weight z-score and lower decrease in HC z-score at discharge compared with PF-fed infants. No differences in weight z-scores were found in VLBW preterm infants fed the different feeding types^(30)^. The differences in z-scores outcomes between the studies could be due to several reasons, including studied populations (non-SGA, VLBW preterm infants), differences in the breast milk type (MOM or donor) and the amount of breast milk (exclusive or any). Our research demonstrates that SGA preterm infants have a unique growth pattern that is affected by feeding type, and expressed by length and not only by weight and HC.

We demonstrated that feeding SGA preterm infants from birth with MOM was associated with poorer length growth at discharge compared with PF feeding. Length is an important indicator of both nutritional adequacy and normal growth. Linear growth is dependent on fat-free mass accretion and adequate protein and micronutrient intake^(31,32)^. Weight measurement alone without length measurements can lead to mis-identification of weight gain or loss, highlighted by previous feedings on preterm infants at term which exhibit less lean and similar fat mass at discharge compared with term infants^(32)^. Furthermore, length was also been associated with brain development and neurodevelopmental outcome^(33,34)^. Recently, expert neonatal dietitians panel recommended using ‘decline in length z-score’ as one of the indicators for identifying neonatal malnutrition or delayed growth in preterm infants^(16)^. Accordingly to this, our study findings showing poorer length growth at discharge of SGA preterm infants fed MOM compared with PF along may indicate signs of malnutrition that could require further enrichment of MOM during hospitalisation, although this could pose a challenge due to the current nutritional protocol. Alternatively, length should be monitored routinely, however should not be a major indicator to malnutrition in this special group of SGA preterm infants and warrant more research.

At 2-year CA, both groups exhibited similar increases in weight and HC z-scores. However, the change in height z-score was different between the two groups, possibly suggesting that the improvement in weight and HC growth of the MOM-fed group was at the expense of height growth. This indicates possible prolonged effects of feeding type on height growth potential. Similarly, Toftlund *et al.* reported in a small sub-cohort that PF-fed SGA preterm infants exhibit rapid growth, whereas MOM-fed (with or without fortifier) SGA preterm infants experienced an increase in weight and height over a longer period of time^(20)^. A recent large observational study on SGA and non-SGA VLBW preterm infants demonstrated slower growth in Δ weight and HC z-scores at discharge among MOM fed compared with PF fed. However, these differences were not observed at age 10^(35)^. Also, in non-SGA preterm infants, an observational cohort study on two large cohorts (LIFT and EPIPAGE) showed greater loss in weight z-score at discharge in MOM-fed preterm infants compared with PF-fed preterm infants. However, at 2-year CA, weight, height and HC z-scores were significantly higher in MOM fed compared with PF fed^(36)^. The EPIPAGE study also found that breast-feeding at discharge was associated with a lower incidence of short stature at 5 years of age^(37)^. Taken together, although there are differences in methodology between the various studies, it can be concluded that MOM-fed SGA preterm infants achieve weight, HC and height goals over longer period than FP-fed SGA preterm infants. However, the question related to the time frame taken to achieve this catch-up is still not entirely clear and depends on feeding type and birth status.

In our study, at 2-year CA the height difference still remained, whereas the PF group exhibited significant linear catch-up growth compared with the MOM group. Poor catch-up growth is affected by prematurity, SGA status, genetics and nutrition and is associated with low IQ and short stature^(38)^. Possible explanation for the difference in height catch-up growth is the amount of protein in the diet. It has been reported that protein content in human milk decreases throughout lactation and varies between mothers^(39,40)^. Also, human milk contains less protein compared with formula and might contribute to slower growth rate^(40)^. According to the ‘early protein hypothesis’, the higher protein content in infant formula compared with human milk could stimulate the production of Insulin growth factor (IGF)-I and insulin which promotes growth^(41)^. In later infancy (∼ 9 months of age), term infants who were still breastfed showed lower levels of IGF-I, IGFBP-3 and insulin than infants no longer breastfed^(42,43)^. Nevertheless, it is important to mention that MOM is the gold standard for feeding preterm infants. Therefore, the question is whether it is right to refer growth on formula as the optimal growth since preterm infants have achieved a greater improvement in height z-score and catch-up growth or rather to refer growth on MOM as the preferred growth in which height catch-up growth occurs over a longer period of time.

In addition, our study demonstrates that feeding SGA preterm infants with fortified MOM from birth was significantly associated with slow increase of HC z-score growth compared with PF feeding at discharge. Studies have shown that poor HC growth in SGA preterm infants may lead to neurodevelopmental impairments outcome^(44,45)^. Although the increase was slower in MOM group, still 98 % of the SGA preterm infants in the study were in the normal range, which may predict positive neurodevelopmental outcomes.

At 2-year CA, the increase in HC z-score was similar in both groups, with PF-fed group showing non-significant trend to higher rate of HC z-score catch-up. Thus, feeding type probably has no effect on HC growth at 2-year CA.

Regarding the nutritional gastrointestinal feeding acceptability, during hospitalisation, significantly less episodes of regurgitation were observed in the MOM group than in the PF group, presumably due to the unique MOM composition which includes digestive enzymes, growth factors, hormones and prebiotic factors which benefit the immature gastrointestinal functions^(46)^. Even though there were more episodes of regurgitation among the PF group, our findings indicate that it did not affect their growth compared with the MOM group.

Our study has several limitations: the feeding information during hospitalisation and post-discharge until the age of 3 months was controlled and thus is detailed and extensive. However, other un-recognised nutritional factors in the feeding post-discharge could have influenced growth. Additionally, parents have fulfilled the questionnaires several years after discharge. Our study has several advantages: we studied a unique population that is hardly investigated, the study population data were fully documented and we studied all the anthropometric parameters. Also, KMC’s NICU follows a standardised and controlled feeding protocol with the same medical staff and dietitian over the years which reduce bias.

In summary, our study points to a link between the feeding type and the rate of linear catch-up growth at discharge and at 2-year CA. SGA preterm infants warrant special attention and closer monitoring of their nutrition before and after discharge to support growth, particularly in MOM-fed infants. Furthermore, supporting earlier birth weight regain should be the focus. This might assist in increasing the height z-score at 2-year CA in MOM-fed SGA preterm infants. Further research is needed to determine the best approach to fortify MOM or to set specific standards to this population in order to achieve optimal growth of SGA preterm infants during the NICU period and at post-discharge period.

### Conclusions

The early growth of the SGA preterm infants is regulated by complex interplay between genetic, environmental, nutritional and endocrine factors, coupled with therapeutic interventions. Our findings indicate that feeding type in SGA preterm infants has an effect on growth in all anthropometric parameters at discharge, whereas at 2-year CA the effect remains only on height growth. Feeding with fortified MOM during hospitalisation compared with PF leads to significant larger decrease in weight and length z-scores and smaller increase in HC z-score at discharge. Feeding SGA preterm infants with unfortified MOM for at least 3 months post-discharge is associated with slower increase in height z-score and delayed height catch-up growth at 2-year CA, suggesting possible prolonged effects on growth potential influenced by feeding type.

Due to differences found in this study, close nutritional monitoring of SGA preterm infants beyond the age of 2 years is warranted.
